# Charge‐Transport Anisotropy in a Uniaxially Aligned Diketopyrrolopyrrole‐Based Copolymer

**DOI:** 10.1002/adma.201502437

**Published:** 2015-10-19

**Authors:** Sam Schott, Eliot Gann, Lars Thomsen, Seok‐Heon Jung, Jin‐Kyun Lee, Christopher R. McNeill, Henning Sirringhaus

**Affiliations:** ^1^ Cavendish LaboratoryUniversity of CambridgeJ J Thomson AvenueCambridgeCB3 0HEUK; ^2^ Department of Materials Science and EngineeringMonash UniversityClaytonVictoria3800Australia; ^3^ Australian Synchrotron800 Blackburn RoadClaytonVictoria3168Australia; ^4^ Department of Polymer Science & EngineeringInha UniversityIncheon402‐751South Korea

**Keywords:** alignment, field‐effect transistors, organic electronics, shearing

## Abstract

**Aligned films of a semiconducting DPP‐based copolymer** exhibit highly anisotropic charge transport with a band‐like temperature dependence along the alignment direction and hole mobilities of up to 6.7 cm^2^ V^−1^ s^−1^. X‐ray diffraction measurements reveal an exceptional degree of in‐plane alignment, high crystallinity, and a dominant face‐on orientation of the polymer backbones. The surprising charge‐transport properties are interpreted in a tie‐chain model consistent with anisotropic activation energies.

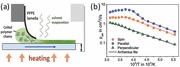

Solution processable semiconducting polymers have recently experienced a boost in performance with charge carrier mobilities in field‐effect transistors (FETs) exceeding 1 cm^2^ V^−1^ s^−1^ in several families of donor–acceptor (D‐A) copolymers.^1^ These polymers have become viable candidates not only for applications in flexible displays and electronics, but also for other devices that rely on efficient charge transport such as organic solar cells and light emitting diodes. Some of the highest performing donor–acceptor polymers known today are based on diketopyrrolopyrrole (DPP) electron accepting units with reported FET mobilities exceeding 1 cm^2^ V^−1^ s^−1^ for both hole‐transport and electron‐transport.^2^, ^3^, ^4^, ^5^, ^6^ D‐A copolymers exhibit a relatively complex backbone structure comprised of alternating electron deficient and electron rich units. They frequently defy a trend established with semicrystalline polymers such as poly(3‐hexylthiophene‐2,5‐diyl) (P3HT) and poly(2,5‐bis(3‐alkylthiophen‐2‐yl)thieno(3,2‐b)thiophene) (pBTTT)^7^, ^8^ of high carrier mobilities being correlated with a high degree of crystallinity. Many high mobility D‐A copolymers show less crystalline, in some cases near amorphous structures. Important factors for the excellent charge transport properties of these materials are a close π–π stacking distance around 3.7 Å,^9^, ^10^, ^11^ a high molecular weight ensuring a network of tie chains that connect the residual crystalline domains^12^ and a low degree of energetic disorder due to a robust planar backbone conformation without backbone torsion.^13^ However, the predominant face‐on stacking observed in some high mobility systems^9^, ^14^, ^15^ and first reports of a band‐like temperature dependence of the field effect mobility above 200 K^16^, ^17^ are examples of the surprising physical properties of these polymers that remain to be fully understood.

An attractive method to improve charge transport in liquid crystalline (LC) polymers is via the introduction of long‐range order by macroscopic in‐plane alignment of polymer backbones.^18^ In‐plane alignment not only enhances mobilities along the preferential backbone direction but also provides a unique opportunity to study the interplay of film morphology and charge transport. The resulting anisotropy of charge transport has played an important role in demonstrating the influence of relative orientations between crystalline grains,^19^ discriminating between interchain and intrachain transport when polymer chains provide sufficient connectivity between crystallites^12^, ^20^ and probing the influence of anisotropic molecular packing in molecular single crystals.^21^

Here we study the charge transport properties of uniaxially aligned films of poly[[2,5‐bis(2‐octadecyl)‐2,3,5,6‐tetrahydro‐3,6‐diketopyrrolo[3,4‐c]pyrrole‐1,4‐diyl]‐alt‐(2‐octylnonyl)‐2,1,3‐benzotriazole] (DPP‐BTz), a recently developed ambipolar DPP‐benzotriazole copolymer with mobilities in spin‐coated, top gate FETs of around 2.5 cm^2^ V^−1^ s^−1^ and a semicrystalline microstructure with pronounced face‐on preferential orientation of polymer chains with respect to the substrate.^22^ The chemical structure of DPP‐BTz is shown in **Figure** **1**a. The combination of a dithienyl‐DPP unit with weak electron‐deficient moieties such as benzothiadiazole (BT) or benzobisthiadiazole (BBT) has proven to yield high performing ambipolar polymers.^14^ With DPP‐BTz, we followed this design route by using benzotriazole (BTz) as comonomer. Even though challenging to synthesize, this molecular design allows for an additional alkyl side‐chain to be attached to the BTz comonomer and permits the tuning of morphology and charge transport through side chain engineering. As discussed by Gruber et al., attaching linear side‐chains to the DPP unit and a branched side‐chain to the BTz unit both significantly changes the conformation of polymer chains and improves charge carrier mobilities.^22^

**Figure 1:**
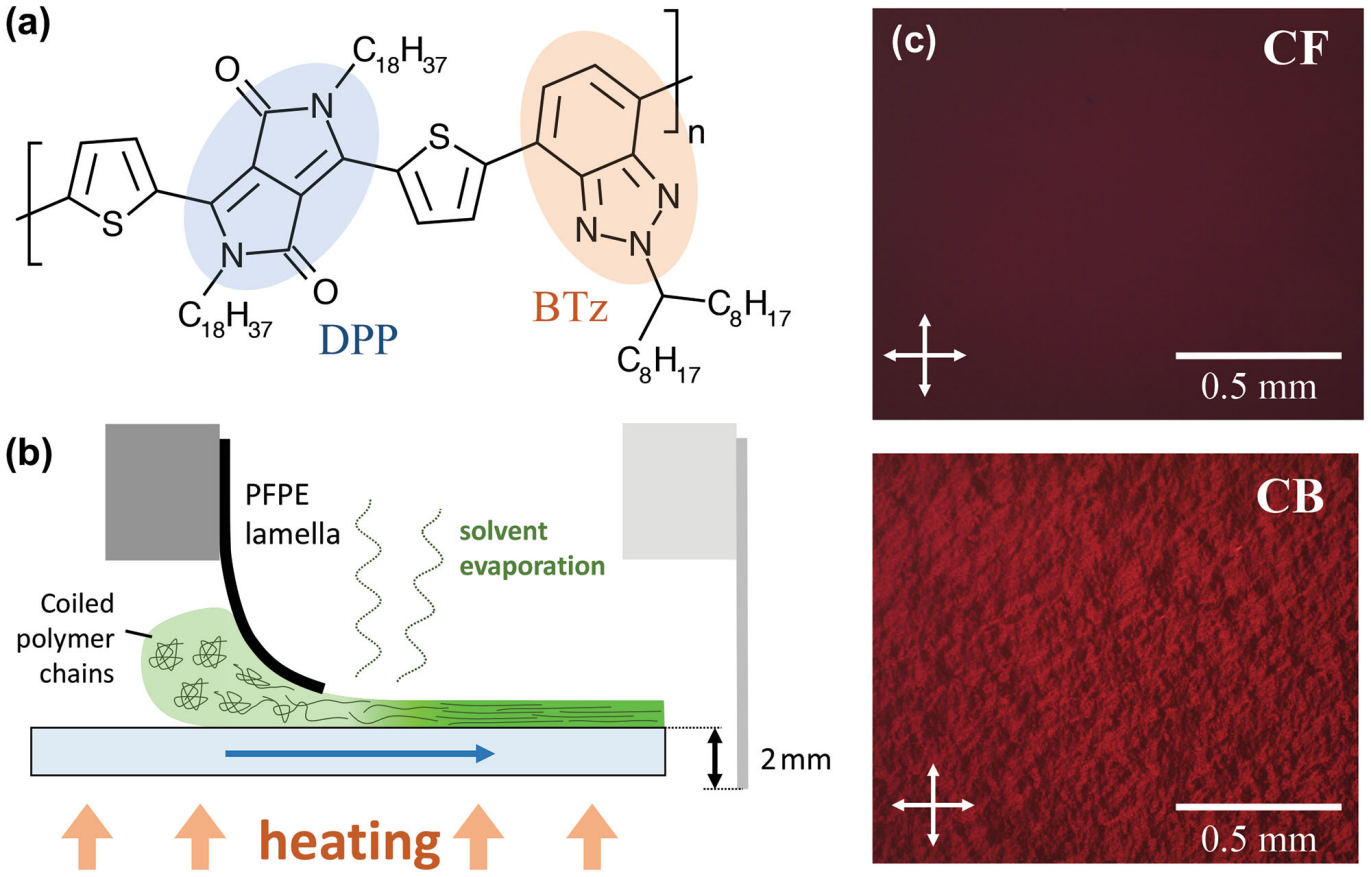
a) Chemical structure of DPP‐BTz. b) Schematic of shearing setup. The sample is fixed on top of a heated stage and can be moved beneath the lamella at speeds from 73 μm s^−1^ to 1.75 mm s^−1^. c) Optical microscopy images of DPP‐BTz films spun from chloroform (CF) and chlorobenzene (CB). The samples are placed between two perpendicular polarizers (indicated by arrows on images). Crystallites with polymer backbones parallel to one of the polarizers remain dark while those at 45° are brightest. The top image (CF) was taken with a longer exposure time to capture a sufficient amount of light.

There are several established methods to induce alignment in conjugated polymer films, based on either the application of macroscopic forces or the use of prestructured template layers. Among the templating methods, two prominent techniques are spin‐coating on top of a rubbed polyimide layer^23^, ^24^ or drop‐casting on a nano‐grooved SiO_2_ surface.^25^, ^26^ Methods based on the application of macroscopic forces include dip‐coating^27^, ^28^, ^29^ and zone‐casting,^30^, ^31^, ^32^, ^33^ which have been shown to induce high degrees of alignment for small molecules, rubbing the polymer film itself^34^, ^35^ and solution‐shearing.^36^, ^37^, ^38^, ^39^ While techniques employing only low shearing forces proved unsuccessful in aligning DPP‐BTz, we present here a newly developed shearing process based on a flexible, solution resistant lamella made of perfluoropolyether (PFPE). This delivered smooth and uniform films with high uniaxial polymer chain alignment that allowed us to study the charge transport anisotropy in this polymer with very high carrier mobilities up to 6.7 cm^2^ V^−1^ s^−1^ along the chain direction.

DPP‐BTz was deposited from chlorobenzene (CB) solutions (10 mg mL^–1^) on top of glass substrates either through a standard spin‐coating process or by shearing with a flexible lamella made from photocured PFPE with the substrate fixed on top of a heated sample stage. Only 7 μL of solution are required to cover a 15 mm × 15 mm substrate by solution shearing compared to 30–50 μL that are necessary to insure uniform coverage in a standard spin‐coated process. A schematic of the setup is depicted in Figure 1b and further details can be found in Section A of the Supporting Information.

The main parameters determining film uniformity and the degree of alignment were shearing speed, substrate temperature and the choice of solvent. DPP‐BTz shows characteristics of a lyotropic LC polymer with the size of crystallites in a cast film strongly depending on the amount of preaggregation induced by a solvent.^40^ This becomes evident when comparing films spin‐coated from different solvents beneath a cross‐polarizing optical microscope (Figure 1c): using chloroform, a solvent known for suppressing the formation of aggregates,^40^, ^41^ no crystalline structure is discernible while films spun from CB exhibit dark and bright patches that correspond to crystallites that are either tilted (bright) or parallel to one of the polarizers (dark).

For the shear alignment process we therefore used CB as solvent. The solvent evaporation rate is set by the stage temperature (fixed at 60 °C) and the boiling point of CB at 131 °C and the degree of alignment is then determined by adjusting the shearing speed. The degree of alignment was quantified by the dichroic ratio $R=A_{∥}/A_{⊥}$ in polarized UV–vis absorption spectroscopy where $A_{∥}$ and $A_{⊥}$ denote the peak absorbance at either the 0–0 or 0–1 vibrational peak. Note that the optical transition dipole moment (TDM) is likely to have a nonzero component perpendicular to the polymer backbone^42^ which implies that *R* only provides a lower bound for the degree of alignment, i.e., *R* would remain finite even in case of perfectly aligned polymer chains.

Dichroic ratios show peak values between 110 and 140 μm s^−1^ (**Figure** **2**a) with a maximum of *R* = 14 ± 1 for 102 μm s^−1^ and dichroic ratios decreasing for higher shearing speeds to *R* = 3.8 ± 0.3 at 292 μm s^−1^. Even though DPP‐BTz presumably has a significant TDM component perpendicular to the backbone due to its monomer shape, the achieved dichroic ratios are among the highest reported in absorption; more typical values for aligned conjugated polymers range between 6 and 12.^18^, ^20^, ^23^, ^24^, ^34^, ^43^ To better compare the degree of alignment determined by polarized absorption with results from grazing‐incidence wide‐angle X‐ray scattering (GIWAXS) and near edge X‐ray absorption fine structure (NEXAFS) measurements, we calculated the 2D structural order parameter:^44^

(1) $$S=〈\cos2\phi 〉=2〈\cos^{2}\phi 〉-1$$

where ϕ is the in‐plane angle between a polymer chain segment and the direction of alignment and the angular brackets denote the average over all chain segments in the film. It can be easily seen that *S* = 0 for isotropic orientations and *S* = 1 for complete uniaxial alignment. Under the assumption that TDMs are oriented parallel to the polymer backbone and noting that the absorption of polarized light scales as $A_{∥}\propto 〈\cos^{2}\phi 〉$ and $A_{⊥}\propto 〈1-\cos^{2}\phi 〉$, the 2D structure parameter can be expressed in terms of the dichroic ratio as $S_{\mathrm{PA}}=\left(R-1\right)/\left(R+1\right)$.^45^ Respective values are given in Figure 2a on the secondary axis and reach their maximum with *S*_PA_ = 0.87. An overview of 2D order parameters extracted from UV–vis, NEXAFS, and GIWAXS is given in **Table** **1**.

**Table 1:** 2D structural order parameters extracted from UV–vis, NEXAFS, and GIWAXS measurement for samples sheared at 102 μm s^−1^

	NEXAFS	UV–vis	GIWAXS
2D order parameter *S*	0.6	0.87 ± 0.01	0.91 − 0.95
Dichroic ratio *R*	4.0	14 ± 1	159 ± 7

**Figure 2:**
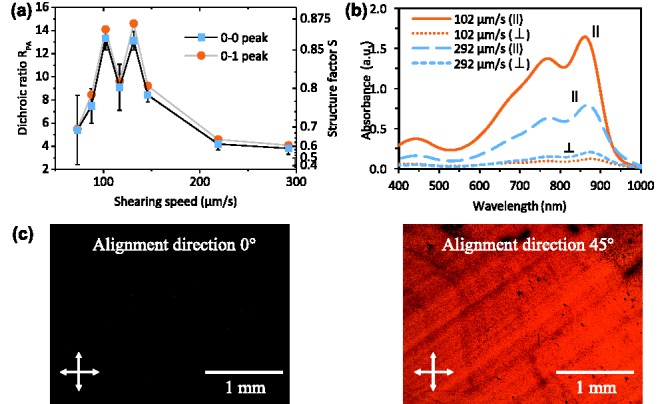
a) Dependence of the dichroic ratio *R* and corresponding 2D structure parameters on the shearing speed for a series of polymer films deposited on glass substrates (conditions as described in the text). Values were averaged from measurements on different spots on the same sample and the dip at 121 μm s^−1^ can be attributed to nonuniform coverage of the sample. b) Absorption spectra of aligned films with 102 and 292 μm s^−1^ shearing speed. c) Cross‐polarizing optical microscope images of film sheared at 102 μm s^−1^, arrows denote directions of polarizers. Both images were taken under identical exposure and light conditions with the film rotated relative to polarizers.

We suspect that the high degree of orientation of polymer chains is facilitated by highly planar backbones. Hydrogen–oxygen interactions between DPP and its adjacent thiophene units are known to increase backbone planarity with density functional theory predicting only small dihedral angles between DPP and its neighbours.^46^ As Kim et al. proposed for sulfur–fluorine interactions between adjacent units, such intra­molecular interactions promoting chain planarity combined with the use of bulky side chains promote lyotropic LC behavior in conjugated polymers.^18^

The decrease of dichroic ratios with higher shearing speeds initially seems counter‐intuitive since a larger flow‐field should result in better alignment. This decrease of *R* most likely stems from the position of film formation relative to the shearing lamella: the solvent evaporation rate is fixed by boiling point and stage temperature so that the point of film formation/drying moves away from the lamella with increasing speeds. This gives chains more time to reorganize again in solution after experiencing the shear force and results in weaker alignment. At speeds below 100 μm s^−1^ however, a significant part of the solvent already evaporates before the shearing process, resulting in nonuniform films or hampering film formation. This manifests itself in smaller dichroic ratios due to nonuniform coverage of samples and higher error bars from higher signal‐to‐noise ratios. Surface profiles from atomic force microscopy (AFM) given in Figure S1, Supporting Information, accordingly show a larger surface roughness. By optimizing the evaporation rate through the choice of solvent and substrate temperature it may be possible to improve the deposition speed of the process for practical applications.

Polarized absorption is an easily accessible and powerful technique for probing the electronic structure and degree of alignment. But it allows only indirect access to the orientation and stacking of polymer backbones. GIWAXS directly probes stacking distances and the morphology via the diffraction of X‐rays by crystalline regions of the film.^47^ Measurements were performed on a spin‐coated reference film and a film sheared at 102 μm s^−1^, both on top of glass substrates. Details of the setup and measurement conditions are given in Section C of the Supporting Information.

2D scattering patterns (**Figure** **3**) show a very pronounced face‐on orientation of backbones with five visible orders of alkyl stacking peaks in the in‐plane direction and a sharp π‐stacking peak in the out‐of‐plane direction. The close π–π stacking distance of 0.38 ± 0.01 nm and the alkyl stacking of 3.0 ± 0.1 nm (extracted from the peak spacing *Q*_hkl_ via $d_{\mathrm{hkl}}=2\pi /Q_{\mathrm{hkl}}$) is comparable to values from other high mobility D‐A copolymers.^1^ The coherence length *τ* of alkyl spacing was estimated from the radial full width at half maximum (FWHM) of the first alkyl stacking peak via Scherrer's equation $\tau =2\pi /Q_{\mathrm{FWHM}}$ with *Q*_FWHM_ in nm^−1^.^47^ The many orders of alkyl‐stacking peaks along the *Q*_xy_ axis and the large alkyl‐stacking coherence length of 31 ± 1 nm confirm an exceptional degree of crystallinity and face‐on anisotropy for this DPP copolymer.

**Figure 3:**
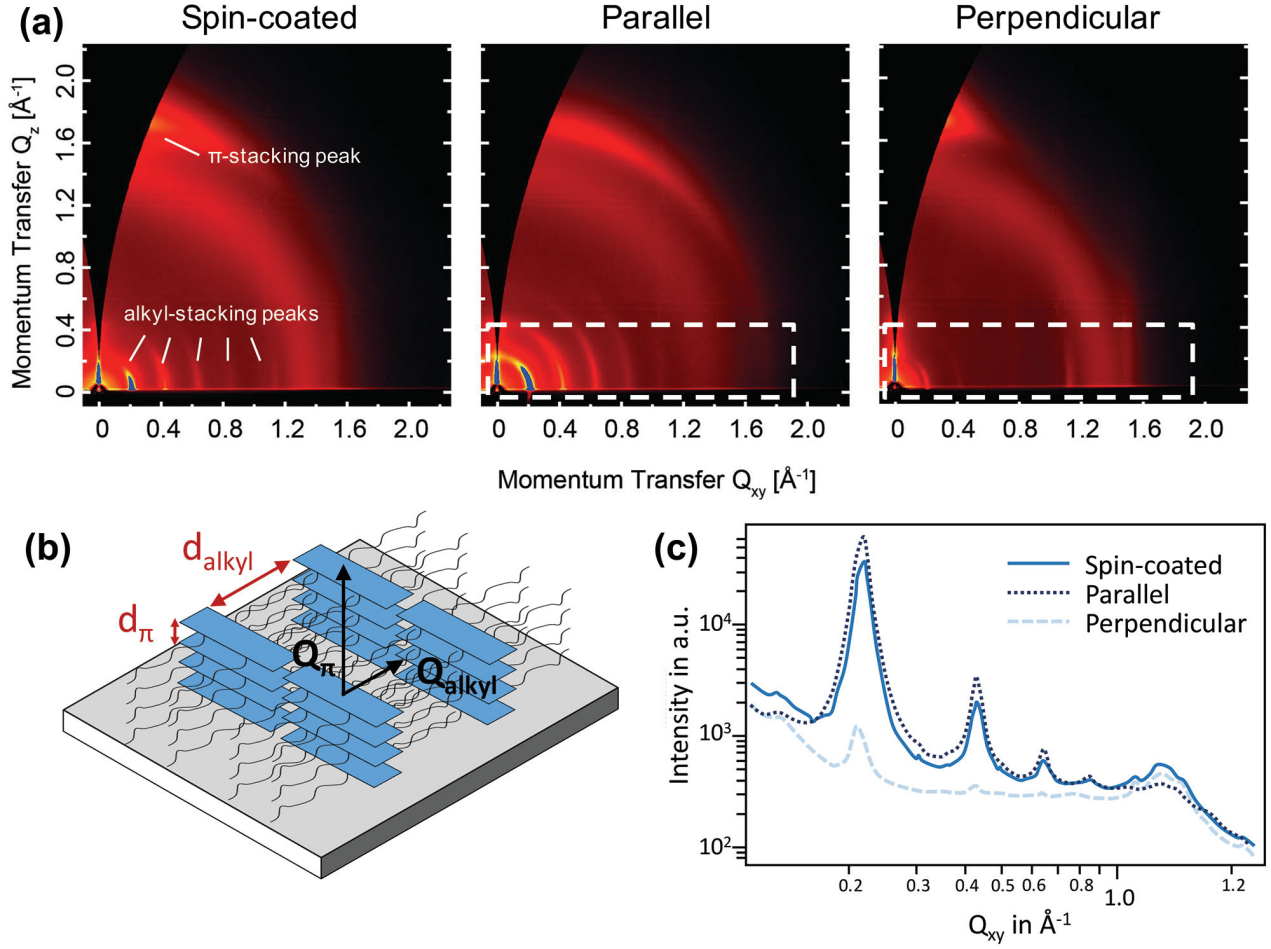
a) Grazing incidence wide‐angle X‐ray scattering patterns of spin‐coated and sheared film (parallel and perpendicular to alignment). b) Schematic of face‐on backbone orientation. c) Cross‐sections of recorded scattering intensities at *Q_z_* = 0.

An overview over extracted spacing and coherence lengths is given in **Table** **2**. Within the accuracy of our measurements, they remain the same for the sheared and spin‐coated samples. Aligned films are therefore likely to exhibit the same chain conformation and packing as spin‐coated films with an absence of lattice strain or other nonequilibrium effects that were observed for zone‐casted TIPS‐pentacene.^38^ The most distinct feature of the 2D scattering patterns is the disappearance of almost all alkyl stacking peaks apart from the first order at *Q*_xy_ ≈ 0.2 Å^−1^ in the measurement perpendicular to the alignment direction. This is expected for a pronounced uniaxial, face‐on chain orientation in which X‐rays impinging at grazing incidence normal to the chain alignment direction are not scattered by the periodicity associated with side chain lamellae. An additional peak at *Q*_xy_ ≈ 1.15 Å^−1^ (spacing of 0.55 nm) increases in intensity with the perpendicular measurement. It can most likely be assigned to the backbone repeat unit and thus confirms the uniaxial chain‐alignment.

**Table 2:** GIWAXS spacing parameters for spin‐coated and sheared samples. π‐stacking distances are calculated from the in‐plane peak at *Q_xy_* ≈ 0.2 Å^−1^

Sample	π‐stacking (out of plane)		Alkyl spacing (in plane)	
	Spacing [nm]	Coh. length [nm]	Spacing [nm]	Coh. length [nm]
Spin‐coated	0.38 ± 0.01	7.0 ± 0.3	2.95 ± 0.04	31.6 ± 0.2
Parallel	0.38 ± 0.01	7.0 ± 0.5	2.97 ± 0.06	30.3 ± 0.2
Perpendicular	0.38 ± 0.01	7.1 ± 0.5	3.03 ± 0.05	35.6 ± 0.5

To quantify the orientation of crystallites in the polymer film in terms of the 2D order parameter, we compare the integrated scattering intensities of the first alkyl stacking peak from parallel and perpendicular measurements, normalized by the alignment independent π‐stacking peak intensity of the respective measurements to compensate for changing scattering geometries (see Table 2). This yields a 159 ± 7 times higher scattering intensity parallel to the alignment direction. Those intensities however do not allow the extraction of a 2D structure factor since they probe the number of crystallites oriented at a specific angle only. To obtain an estimate for the structure factor we approximate the distribution of orientations around the alignment direction I(ϕ) with a Gaussian function on top of a constant background in agreement with previously measured azimuthal scans of scattering intensities.^45^, ^48^ The ratio between peak and background is determined by the scattering intensities at 0° and 90° and we conservatively calculate with a FWHM between 10° and 40° compared to a FWHM < 10° for aligned P3HT.^45^, ^48^ The order parameter can then be calculated from:^48^

(2) $$〈\cos^{2}\phi 〉=\frac{\int_{-\pi /2}^{\pi /2}I\left(\phi \right)\cos^{2}\left(\phi \right)d\phi }{\int_{-\pi /2}^{\pi }I\left(\phi \right)d\phi }$$

The nomenclature I(ϕ) was chosen to emphasize that the distribution is in principle proportional to the scattering intensity after careful rescaling to account for changes in scattering geometries. The resulting value span of *S*_GIWAXS_ = 0.91–0.95 demonstrates an exceptional orientation of crystallites. The slightly lower order parameter estimated from polarized absorption probably stems from including amorphous and crystalline regions as well as the effect of off‐axis transition dipoles perpendicular to the polymer chain axis. The degree of alignment is, to the best of our knowledge, among the highest achieved for semiconducting polymers.

Near edge X‐ray absorption fine structure spectroscopy probes the angle dependent absorption of linearly polarized X‐rays into the C1s‐π* resonance.^49^, ^50^ Unlike GIWAXS, it is sensitive to both crystalline and amorphous regions and allows for the extraction of the average orientation of polymer backbones due to the orientation of C1s‐π* TDMs normal to the conjugation plane (**Figure** **4**c). X‐rays are polarized linearly in the plane of incidence and by scanning over different azimuthal angles π or tilt angles *θ* of the incident X‐ray beam, it is possible to determine the degree of uniaxial in‐plane alignment and the average tilt of conjugated planes with respect to the substrate normal, respectively.

**Figure 4:**
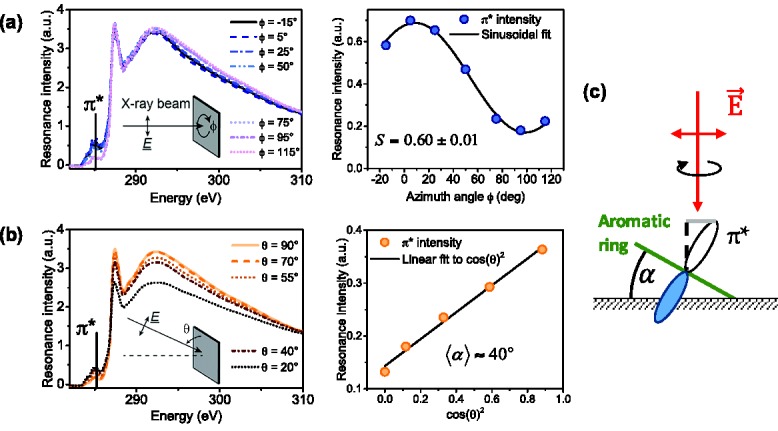
a) TEY spectra from azimuthal angle scan of sheared film and sinusoidal fit to C1s‐π* resonance intensity for extraction of dichroic ratio. The offset of 5° for π is caused by the inprecision of placing the alignment direction along the horizontal axis. b) TEY spectra from tilt angle scan of sheared film and fit to π* resonance intensity for extraction of average backbone tilt. c) Contribution of TDMs to resonance intensity for azimuthal scan at θ = 90°.

To determine the degree of in‐plane alignment, we keep the X‐ray beam fixed at normal incidence (*θ* = 90°) and scan over azimuthal angles ϕ, thereby effectively rotating the polarization around the surface normal (Figure 4c).

The degree of alignment is again quantified in terms of the 2D order parameter $S_{\mathrm{NEXAFS}}=(I_{\max}-I_{\min})/(I_{\max}+I_{\min})$ which corresponds to the percentage difference between maximum and minimum resonance intensities *I*_max_ and *I*_min_ that are achieved perpendicular and parallel to the alignment direction, respectively. A derivation of *S*_NEXAFS_ can be found in Section D of the Supporting Information.

DPP‐BTz was deposited under similar conditions as for GIWAXS but on top of heavily n‐doped Si substrates to prevent charging effects. Due to the different wetting behavior of doped Si, insights on film morphology must be treated with some caution when comparing FET performance with polarized NEXAFS measurements.

The π* resonance intensity in total electron yield mode (TEY) peaks at ϕ=0° with the X‐ray polarization parallel to the C1s‐π* transition dipole moment and perpendicular to the direction of alignment and reaches its minimum at ϕ=90° parallel to alignment (Figure 4a). TEY mode measures both Auger and secondary electrons and is sensitive to the top ≈3 nm of the film where the electron kinetic energy is sufficient to escape the surface. The resulting order parameter of $S_{\mathrm{NEXAFS}}=0.6$ confirms a significant degree of in‐plane alignment, albeit smaller than previously achieved values for zone‐cast PBTTT (*S*_NEXAFS_ = 0.74)^51^ and N2200 (*S*_NEXAFS_ = 0.86).^20^ This reduced in‐plane order compared to optical and GIWAXS observations could indicate a reduced degree of order at the surface, or that samples prepared on silicon had reduced order compared to samples prepared on glass. Bulk‐sensitive fluorescence yield (FY) measurements unfortunately were too noisy to extract a reliable dichroic ratio.

Tilt angle scans revealed a distinct change in morphology toward the film surface in both sheared and spin‐coated films. The top 1 nm surface layer, accessible in partial electron yield NEXAFS, is most likely covered in insulating alkyl side‐chains with almost negligible π* resonance intensities (Figure S3, Supporting Information). Such an insulating layer on the sample surface should be favorable for charge transport by shielding the accumulation layer from dielectric induced polar disorder. FET performance does indeed increase when switching from CYTOP (*ε*_r_ = 2.1) to PMMA (*ε*_r_ = 3.6) dielectrics even though the higher dielectric constant causes increased polar disorder.

π* resonances reappear in TEY (Figure 4b), sensitive to the top 3 nm, and increase in bulk sensitive fluorescence yield mode. Fitting the tilt angle dependence of the π* resonance intensity to Equation S1 (Section D of the Supporting Information) allows the extraction of backbone tilt angles as $〈\cos^{2}\alpha 〉=0.59$ and $〈\cos^{2}\alpha 〉=0.81$ for the surface layer and bulk film, respectively ($〈\cos^{2}\alpha 〉=1$ denotes fully face‐on backbones). This corresponds to average tilt angles of 〈α〉≈40° and 〈α〉≈26° for the surface layer and bulk film. Note that even though an average angle can be extracted, its value gives only limited insight into the distribution of orientations around this angle. This dominant face‐on structure, with a stronger tilt of backbones toward the surface layer, positions DPP‐BTz among the most face‐on semiconducting polymer reported so far and exceeds values for indacenodithiophene‐co‐benzothiadiazole (IDT‐BT), considered one of the most face‐on polymers, with $〈\cos^{2}\alpha 〉$ between 0.57 and 0.67.^15^ Extracted tilt angles are the same for spin‐coated and aligned films, again confirming that we do not change the microstructure but only reorient the semi‐crystalline domains during shearing.

The difference in surface morphology, together with *S*_NEXAFS_ < *S*_PA_ indicates a weaker alignment of polymer chains at the film surface. At this point, it is unclear whether the distinct surface morphology develops during the deposition process or is caused by a lower glass transition temperature *T*_g_ at the film surface (the surface value of *T*_g_ can be up to 80 °C lower than the bulk value^52^). Performing the same measurements on delaminated films to access the morphology of the buried interface layer^53^ should allow the exclusion or confirmation of glass transition effects. Furthermore, it should be noted that the contribution of TDMs to the resonance intensity when probing in‐plane alignment at normal incidence increases for stronger backbone tilts and disappears for fully face‐on backbones (see Figure 4c). The 2D order parameter extracted from NEXAFS is more sensitive to chains with edge‐on backbones than those with face‐on backbones and may therefore be considered a less accurate probe of chain alignment in our predominantly face‐on oriented films than optical absorption or GIWAXS.

The above shearing process was used to manufacture FETs with aligned polymer films. DPP‐BTz was deposited from a CB solution (10 mg mL^−1^) on top of substrates with evaporated gold source–drain electrodes (channel angles from 0° to 90° relative to shearing direction and a channel length of *L* = 20 μm). Preceding dielectric and gate deposition, the samples were annealed at 110 °C for 1 h inside a N_2_ glove box and a 500 nm layer of poly(methyl methacrylate) (PMMA) was spin‐coated on top as a gate dielectric. FETs were manufactured with shearing speeds from 102 to 730 μm s^−1^. Polymer films from lower shearing speeds were too uneven and patchy to produce reliably working transistors and were therefore excluded from these analyses.

Saturated field‐effect mobilities (*μ*_sat_) were determined via the slope of a linear fit to *I*_SD_^1/2^ over the gate voltage *V*_G_ in the last 20 V of transfer curves (from −40 to −60 V at *V*_SD_ = −60 V) by:

(3) $$\mu_{\mathrm{sat}}=\frac{2L}{WC_{i}}{\left(\frac{d\sqrt{I_{\mathrm{SD}}}}{dV_{G}}\right)}^{2}$$

with *L* and *W* being the transistor channel length and width, respectively, and *C*_i_ being the gate dielectric capacitance per unit area. Equation (3) is derived for ideal transistor characteristics where the source drain current in the saturation regime reads:

(4) $$I_{\mathrm{SD}}^{\mathrm{sat}}=\frac{W}{2L}\mu C_{i}{\left(V_{G}-V_{\mathrm{th}}\right)}^{2},|V_{\mathrm{SD}}\left|\geq |V_{G}-V_{\mathrm{th}}\right|$$

with the threshold voltage *V*_th_.^54^

The in‐plane alignment clearly results in anisotropic charge transport with increased saturation mobilities measured when transport is along the preferential direction of polymer chains (*μ* ^||^_sat_) and mobilities lower than the spin‐coated average for devices in which the current direction is perpendicular to the alignment direction (*μ* ^⊥^_sat_). The average values of *μ* ^||^_sat_ and *μ* ^⊥^_sat_ for different shearing speeds are given in **Figure** **5**c. Saturation mobility anisotropies are generally lower than the dichroic ratios extracted from absorption spectra with a peak in mobility anisotropy at room temperature for the 102 μm s^−1^ sample of *μ* ^||^_sat_/*μ* ^⊥^_sat_ = 3.1 ± 1.6 (the large uncertainty is owed to the division of two values each with error bounds derived from the statistical distribution of mobility values extracted from different devices). Even though the alignment is strongest for low shearing speeds, those samples exhibit lower mobilities than the maximum values achieved at 131 μm s^−1^, presumably because of defects in the polymer films and a resulting shorter effective channel width. At 131 μm s^−1^, where film quality and alignment are balanced, a remarkable maximum mobility of *μ* ^||^_sat_ = 6.7 cm^2^ V^−1^ s^−1^ was achieved (transfer and output characteristics in Figure 5). Unlike several other reports of mobilities exceeding 5 cm^2^ V^−1^ s^−1^,^3^, ^25^ mobilities extracted for DPP‐BTz were maintained over a much wider gate voltage range of 20 V and up to a high gate voltage of *V*_G_ = −60 V. Extending the range of mobility extraction to 30 V (from −30 to −60 V) yielded only 10–25% lower values, i.e., in spite of the slight nonidealities in the saturated transfer characteristics we consider these high mobilities as robust values.

**Figure 5:**
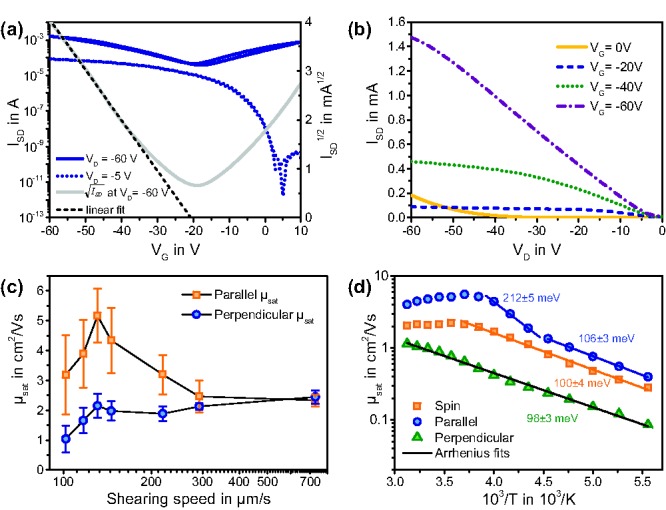
a) Transfer and b) output characteristics of aligned FET with *μ*_sat_ = 6.7 cm^2^ V^−1^ s^−1^. c) Hole mobilities of FETs with aligned polymer layers (room temperature), parallel and perpendicular to shearing direction. Error bars represent the standard deviation over multiple samples. d) Temperature dependence of mobilities for spin‐coated and aligned FETs. Arrhenius fits are labeled with the respective extracted activation energies.

The anisotropy of charge transport can be understood qualitatively by charges being delocalized along the polymer backbone. Since the effective conjugation length along a straight chain segment still is much smaller (≈10 nm) than the channel length, interchain hopping remains the rate‐limiting step even for transport parallel to the direction of chain alignment. However, charges require a smaller number of transport limiting interchain hops to cross the channel when transported along the preferential chain direction. Assuming that hopping distances are much smaller than the average distance travelled along the backbone between hopping events, it has been estimated from a geometric model that the ratio between mobilities should be of the same order of magnitude as the dichroic ratio *R*_PA_ from polarized absorption.^20^ The fact that this ratio is quite a bit smaller for the sheared FETs might be explained by weaker alignment at the accumulation layer than in the bulk material. This is consistent with the lower 2D order paramater for the top ≈3 nm in NEXAFS. Another viable explanation could be that charge transport may be limited by grain boundaries and disordered regions between crystallites while absorption spectra are less sensitive to those regions.

To identify differences in the charge transport mechanisms parallel and perpendicular to polymer chains, we conducted FET measurements at temperatures ranging from 180 to 320 K on both spin‐coated and sheared FETs (131 μm s^−1^). The temperature dependence of mobilities from the sheared and spin‐coated samples (Figure 5d) shows several remarkable features such as different activation energy regimes for sheared samples, peak anisotropies in mobility exceeding a factor of 10 at 250 K and most notably, a cross over to a band‐like temperature dependence of the mobility above 270 K, both for spin‐coated samples and (more pronounced) for devices with transport along the shearing direction.

Between 180 and 270 K an Arrhenius like temperature dependence is observed for all FETs, indicating thermally activated hopping transport as expected for polymeric semiconductors. For *μ* ^⊥^_sat_, this Arrhenius dependence extends up to 320 K and shows an activation energy of *E*_a_ = 98 ± 3 meV, extracted from *μ* ∝ exp(−*E*_a_/*k*_B_ *T*) with the Boltzmann constant *k*_B_ and temperature *T*. This value is higher than activation energies reported for other high mobility polymers with *E*_a_ approaching the polaronic reorganization energy^55^, ^56^ but this might be partially caused by dipolar disorder at the PMMA dielectric interface. While the spin‐coated device exhibits a similar activation energy, transport parallel to the alignment direction has two distinct regions with different *E*_a_ values. At low temperatures from 180 to 230 K, *E*_a_ is of the same size as the spin‐coated and perpendicular devices. But from 230 to 260 K, a significantly higher activation energy of *E*_a_ = 212 ± 5 meV is observed, exceeding values of 50–90 meV for common high mobility polymers like diketopyrrolopyrrole‐co‐ benzothiadiazole (DPP‐BT) or pBTTT.

The most striking feature of the transport properties is the band‐like temperature dependence of μ_sat_ observed above 270 K for the sample in which charge transport is along the chain alignment direction. We confirmed this temperature dependence with transistor characteristics obtained from four different samples and could see only little difference between cooling down and heating up cycles. Joule heating of FETs could be a possible error source even at room temperature that would manifest itself in underestimated activation energies but would not cause a decrease of mobilities with higher temperature. From the weak nonlinearity at the onset of output characteristics (Figure S4, Supporting Information), we expect only small contact resistance effects and cannot see any significant changes with temperature or FET channel direction. It is important to emphasize that in our measurements not only the extracted mobility but also the measured transistor current itself decreases from 270 to 320 K (Figure S5, Supporting Information) which demonstrates that in this temperature range, charge transport indeed improves with lower temperatures and the observed band‐like transport characteristics are not an artefact from the mobility extraction method.

To our knowledge, there are only two previous reports of similar band‐like transport in semiconducting polymers at this time, both in similar D‐A copolymers. The first report from Senanayak et al.^16^ ascribes the band‐like temperature dependence in another DPP copolymer (2DPP‐TEG) to thermally activated hopping into transport states above a mobility edge and argues that this can be suppressed by introducing interfacial disorder with high‐*k* dielectrics. However, they see large fluctuations between similar devices, ranging from near constant mobilities between 150 and 300 K to thermally activated hopping transport up to 300 K, which complicates a consistent interpretation of results. Yamashita et al.^17^ observe both band‐like temperature dependence and a Hall effect in aligned (weakly crystalline) films of the polycyclopentadithiophene‐benzothiadiazole copolymer CDT‐BTz. They see Hall factors of ≈0.5 at 280 K and close to unity above 300 K, indicating stronger localization of charges at lower temperatures, but only probe transport parallel to the alignment direction and above 240 K. They do not draw any conclusions about the relationship between morphology and charge transport but the fact that they align polymer films to achieve band‐like transport suggests improved mobilities compared to conventional spin‐coated samples.

Our results similarly suggest that along the polymer chain direction a band‐like transport regime can be reached at sufficiently high temperatures. In this regime, transport is no longer thermally activated but is adversely affected by thermal fluctuations of the molecular structure/packing or backbone conformation. Within the model developed by Noriega et al., amorphous regions that separate semicrystalline domains are characterised by a larger band‐gap and hinder charge transport.^12^ They are bridged by tie chains that connect neighbouring crystallites and become accessible at higher thermal energies. In case of uniaxial alignment, these tie chains would result in a discrimination between directions: Along the direction of alignment, tie chains can be straight extensions of chains from within crystallites whereas for transport perpendicular to the direction of chain alignment tie chains connecting adjacent crystallites necessarily have to bend and thus exhibit breaks in conjugation. This would suggest that for transport along the chain alignment direction charges encounter less energetic disorder in the amorphous regions between crystallites. For transport perpendicular to the alignment direction, the degree of energetic disorder experienced when crossing these regions is likely to be larger. Accordingly, charge transport remains limited to hopping between tail states in a density of states (DOS) with larger energetic disorder. In contrast, states which constitute efficient transport pathways along extended tie chains and which lie at higher energies in the DOS become thermally accessible for transport along the direction of chain alignment at sufficiently high temperatures. This is also consistent with the steeper increase in parallel mobilities between 230 and 260 K.

The above observations are even more remarkable when considering the strong face‐on orientation of DPP‐BTz with insulating side chains hindering in‐plane hopping between polymer chains. Although the degree of face‐on alignment is not as high on the surface as in the bulk, this clearly shows that the accumulation layer extends over more than one π‐stacking layer so that interchain transport takes place as efficiently as in edge‐on orientations. A similar argument has been proposed for a naphthalenediimide and bithiophene copolymer (NDI‐T2) with strong face‐on morphology^9^ where the annealing induced transition to edge‐on stacking had little effect on charge transport.^57^

Our study demonstrates the alignment, structural and electronic characterisation of a liquid crystalline DPP‐based copoly­mer obtained by attaching linear side‐chains at the DPP unit and a branched sidechain at the BTz comonomer. With a new solution shearing process, we are able to induce a high degree of in‐plane anisotropy with in‐plane order parameters ranging from *S* = 0.87 in UV–vis absorption to *S* = 0.91–0.95 for X‐ray diffraction without introducing lattice strain or changing unit cell parameters. The structural anisotropy is reflected by a charge transport anisotropy with a factor >10 between saturation mobilities parallel and perpendicular to alignment at 250 K. The reduced anisotropy in mobility compared to structural anisotropy in the bulk film can be traced back to a morphology change at the film surface which constitutes the FET accumulation layer, identified by near edge X‐ray absorption spectroscopy. Nevertheless, we are able to achieve maximum values of *μ*_sat_ = 6.7 cm^2^ V^−1^ s^−1^ at room temperature, significantly higher than mobilities in spin‐coated devices and, in fact, one of the highest, robustly extracted mobility values reported for a polymer FET. In these films we have reproducibly been able to observe a band‐like temperature dependence of the mobility in devices in which transport is preferentially along the polymer chain alignment direction. This band‐like transport regime along the polymer chain, in which transport becomes limited by thermal fluctuations, is potentially an important route for further improving the performance of polymer FETs, but will need to be studied in more detail. The nature of the thermal fluctuations is unclear at present; they might be related to torsional motions hindering transport along the polymer backbone but could also be related to thermal lattice fluctuations of the relative positions of adjacent polymer chains modulating the transfer integrals for charge carrier hopping between chains, similar to what is observed in high mobility molecular systems.^58^

## Supporting information

As a service to our authors and readers, this journal provides supporting information supplied by the authors. Such materials are peer reviewed and may be re‐organized for online delivery, but are not copy‐edited or typeset. Technical support issues arising from supporting information (other than missing files) should be addressed to the authors.

Supplementary
