# Supplementary material for: Charge‐Transport Anisotropy in a Uniaxially Aligned Diketopyrrolopyrrole‐Based Copolymer
Source: Adv Mater. 2015 Oct 19;27(45):7356–64. doi: 10.1002/adma.201502437 (PMC4768648; doi:10.1002/adma.201502437)
Supplement: Supplementary file 1 — Supplementary [file ADMA-27-7356-s001.pdf]

Copyright WILEY-VCH Verlag GmbH & Co. KGaA, 69469 Weinheim, Germany, 2015.

ADVANCED MATERIALS

Supporting Information

for *Adv. Mater.*, DOI: 10.1002/adma.201502437

Charge-Transport Anisotropy in a Uniaxially Aligned Diketopyrrolopyrrole-Based Copolymer

*Sam Schott, Eliot Gann, Lars Thomsen, Seok-Heon Jung, Jin-Kyun Lee, Christopher R. McNeill, and Henning Sirringhaus**

Copyright WILEY-VCH Verlag GmbH & Co. KGaA, 69469 Weinheim, Germany, 2013.

Supporting Information

Charge transport anisotropy in uniaxially aligned diketopyrrolopyrrole-based copolymers

Sam Schott,[1)] Eliot Gann,[2),3)] Lars Thomsen,[3)] Seok-Heon Jung,[4)] Jin-Kyun Lee,[4)] Christopher R. McNeill,[2)] and Henning Sirringhaus[1)]*

Sam Schott, Dr. Eliot Gann, Dr. Lars Thomsen, Seok-Heon Jung, A/Prof. Jin-Kyun Lee, A/Prof. Christopher R. McNeill, and Prof. Henning Sirringhaus
[1)]Cavendish Laboratory, University of Cambridge,
J J Thomson Avenue, Cambridge, CB3 0HE (United Kingdom)
[2)]Department of Materials Science and Engineering, Monash University,
Clayton, Victoria 3800 (Australia)
[3)]Australian Synchrotron, 800 Blackburn Road, Clayton, Victoria 3168 (Australia)
[4)]Department of Polymer Science & Engineering, Inha University,
Incheon, 402-751 (South Korea)

A) FET fabrication and testing

Polymer deposition: The PFPE lamella used for solution shearing was manufactured using liquid PFPE-urethane dimethacrylate (Fluorolink® PFPE MD700) as described in[1] and the lamella was kept at a constant height corresponding to 3 mm below the glass substrate surface when straightened. Non-aligned films where spin-coated at 2000 rpm for 90 sec. All films where annealed at 110°C for 1 h after deposition and subsequently quench-cooled on a metal surface at room temperature. Both deposition and annealing were conducted inside a controlled nitrogen environment.

FET fabrication: For the device fabrication, photolithographically defined gold electrodes (20 nm thick) with a chromium adhesion layer (4 nm thick) were evaporated onto glass substrates. After depositing and annealing the polymer film, a 500 nm thick PMMA dielectric film was spin-coated on top from n-butyl acetate at 1500 rpm for 60 sec and annealed at 90°C for 30 min. 25 nm thick gate electrodes (gold) were finally evaporated on top through a shadow mask.

FET measurements: Transfer and output characteristics of FETs were measured using an Agilent 4155B Semiconductor Parameter Analyzer, either inside a nitrogen glove box at room temperature or in a vacuum < 10^{-7} mbar using a cold finger cryostat with heating circuits and liquid nitrogen cooling (Desert Cryogenics Low Temperature Probing Station).

B) AFM surface profiles

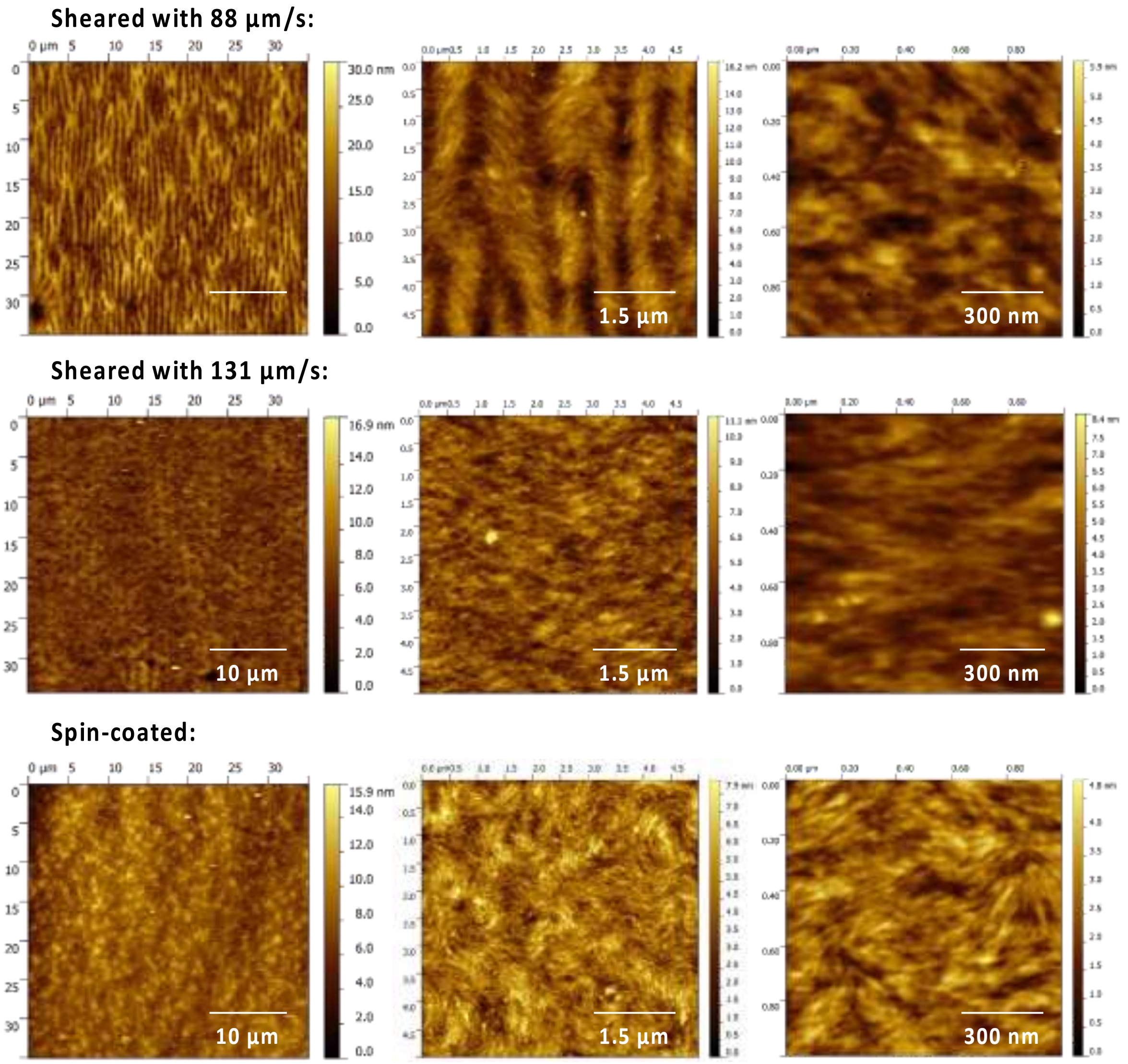

Figure S1. AFM images of representative sheared and spin-coated DPP-BTz films. Shearing direction is from right to left.

Surface profiles recorded with atomic force microscopy confirm the conclusions from UV-Vis spectroscopy. In Figure S1 we show representative DPP-BTz films deposited with sheering speeds of 88 µm s^{-1} and 131 µm s^{-1}. The change in surface roughness and uniformity

is reflected by the root mean square (*RMS*) of the hight profile. The filmed sheared with 88 μm s^{-1} is relatively rough with *RMS* = 2 nm and has clearly visible valleys and hills perpendicular to the shearing direction, the 131 μm s^{-1} film is much smoother with *RMS* = 1.2 nm. Both films exhibit a clear preferential direction with crystalline domains oriented in a wave-like pattern with varying angles towards the shearing direction. This fluctuation is less pronounced but still discernible for the 131 μm s^{-1} film. In contrast, the spin-coated film shows only randomly oriented crystallites with small local correlations in orientation and a comparatively smooth surface with *RMS* = 0.8 nm (*RMS* values have been calculated from the 5×5 μm^2 images in the middle).

C) GIWAXS details

Synchrotron-based grazing-incidence wide-angle x-ray scattering (GIWAXS) measurements of DPP-BTz were performed at the small/wide angle x-ray scattering beamline at the Australian Synchrotron.[2] 9 keV Photons were used with 2D scattering patterns recorded by a Pilatus 1M detector. Scattering patterns were recorded as a function of x-ray angle of incidence, with the angle of incidence varied from 0.05 degrees below the critical angle of the organic film to 0.2 degrees above the critical angle. The images reported were at the critical angle as identified by the angle with the highest scattering intensity. Data acquisition times of 3 sec were used, with three 1 sec exposures taken with offset detector positions to cover gaps in the Pilatus detector. X-ray scattering data is expressed as function of the scattering vector, q, that has a magnitude of $(4\pi/\lambda)\sin(\theta)$, where θ is half the scattering angle and λ is the wavelength of the incident radiation. GIWAXS data was analysed with IGOR using code based on a modified version of Nika.[3]

D) NEXAFS details

NEXAFS measurements were performed at the Soft X-ray Spectroscopy beamline at the Australian Synchrotron.[4] Nearly perfectly linearly polarized photons ($P \approx 1$) from an

APPLEII undulator X-ray source with high spectral resolution ($E/\Delta E \leq 10\,000$) were focused into an ultrahigh vacuum chamber to a ~60 × 400 μm spot size. X-ray absorption was measured using partial electron yield (PEY), total electron yield (TEY) and total fluorescence yield (FY) methods. PEY detection was performed by detecting photoelectrons emitted from the surface with a channeltron detector. TEY detection was performed by measuring the drain current flowing into the sample to neutralize electrons leaving the sample. FY detection was performed using a multichannel plate detector with sufficient retarding voltage to repel all electrons. A fresh spot on the sample was used for each spectrum with the acquisition time minimized (0.5 sec per 0.1 eV step) to prevent beam damage. The recorded signals were normalized to the incident photon flux using the “stable monitor method,” in which the sample signal is compared consecutively to a clean reference sample and the time variation in flux is measured via a gold mesh.[5] The normalized spectra were scaled by normalizing the pre-edge (at 280 eV) to zero and the post-edge (at 320 eV) to one, effectively normalizing to the total carbon content of the material. The photon energy was calibrated by measuring the NEXAFS spectrum of highly oriented pyrolytic graphite simultaneously to the sample signal and normalizing to the exciton peak at 291.65 eV.[6]

The surface sensitivity in NEXAFS depends on the employed detection method. The core hole remaining after excitation by X-rays is highly unstable and is filled either by fluorescent decay or Auger decay, the latter being predominant in low-Z molecules such as organics. The number of fluorescence photons or Auger electrons is proportional to the absorption cross-section, i.e., the projection of the electric field vector along the transition dipole moment. Electrons emitted by Auger decay are scatter in-elastically and release a cascade of secondary electrons from which only those with sufficient kinetic energies escape from the film and contribute to the signal. Thus, TEY detection is only sensitive to the top few nanometers beneath the surface (~3 nm for our samples). By detecting only electrons with a kinetic

energy above a certain threshold, the surface sensitivity can be narrowed even further to ~1 nm in PEY mode. In contrast to electrons, fluorescent photons are rarely scattered within the thin sample and the X-ray penetration depth becomes the only limiting factor. For thin films such as our samples, FY mode is practically bulk sensitive. To prevent charging effects from Auger and secondary electrons, all samples were prepared on highly doped silicon wafers.

For spin-coated films with randomly oriented crystalline domains, there is no preferential in-plane direction. For such a three-fold or higher symmetry, the C1s-π* resonance intensity is given by[7]

$$I \propto \frac{1}{3}\left[1 + \frac{1}{2}(3\cos^2\theta - 1)(3\langle\cos^2\alpha\rangle - 1)\right] \tag{S1}$$

with the angle of X-ray incidence θ and the angle α between substrate plane and conjugated backbone (for coordinate system see Figure S1). $\langle\cos^2\alpha\rangle$ is calculated by fitting Equation (S1) to 1s-π* resonance intensities. Giving an exact value for $\langle\alpha\rangle$ is only possible at the extremes of complete face-on $\langle\cos^2\alpha\rangle = 0$ or edge-on $\langle\cos^2\alpha\rangle = 1$ orientation. At the "magic angle" of $\alpha \approx 54.7°$, Equation (S1) becomes $I \propto \frac{1}{3}$ and it is impossible to distinguish between an isotropic distribution or an orientation of all conjugated planes at precisely the magic angle.

In case of two-fold symmetry such as uniaxial in-plane alignment or when considering a single straight polymer chain segment, the resonance intensity additionally depends on the azimuthal angle ϕ between incident X-ray polarization and the 1s-π* transition dipole moment:[7]

$$I \propto \cos^2\theta\cos^2\alpha + \sin^2\theta\sin^2\alpha\cos^2\phi. \tag{S2}$$

This simplifies to $I_{\theta=90°} \propto \sin^2\alpha\cos^2\phi$ when measuring the in-plane orientation with X-rays incident normal to the substrate plane ($\theta = 90°$). Since we have a distribution of backbone

orientations, we can write $\phi = \phi_j - \phi_{xr}$ with the in-plane orientation of the j-th polymer chain ϕ_j and the azimuthal angle of X-ray incidence ϕ_{xr}. The total resonance intensity then is

$$I_{\theta=90^\circ} \propto \langle \cos^2(\phi_j - \phi_{xr}) \rangle_j. \qquad \text{(S3)}$$

With TDMs predominantly oriented around $\phi_j = 0$ due to alignment, the intensity reaches is maximum at $\phi_{xr} = 0°$ and its minimum at $\phi_{xr} = 90°$. This gives the proportionalities $I_{max} \propto \langle \cos^2 \phi_j \rangle_j$ and $I_{min} \propto \langle \cos^2(\phi_j - 90°) \rangle_j$ and allows us to calculate the 2D order parameter via $\langle \cos^2(\phi_j) \rangle = I_{max}/(I_{max} + I_{min})$ and $S = (I_{max} - I_{min})/(I_{max} + I_{min})$.

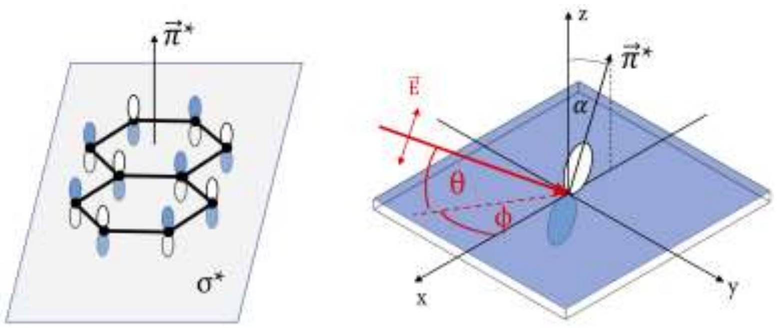

Figure S2. Orientation of 1s-π* transition dipole moment $\vec{\pi}^*$ on polymer backbone and schematic of NEXAFS geometry.

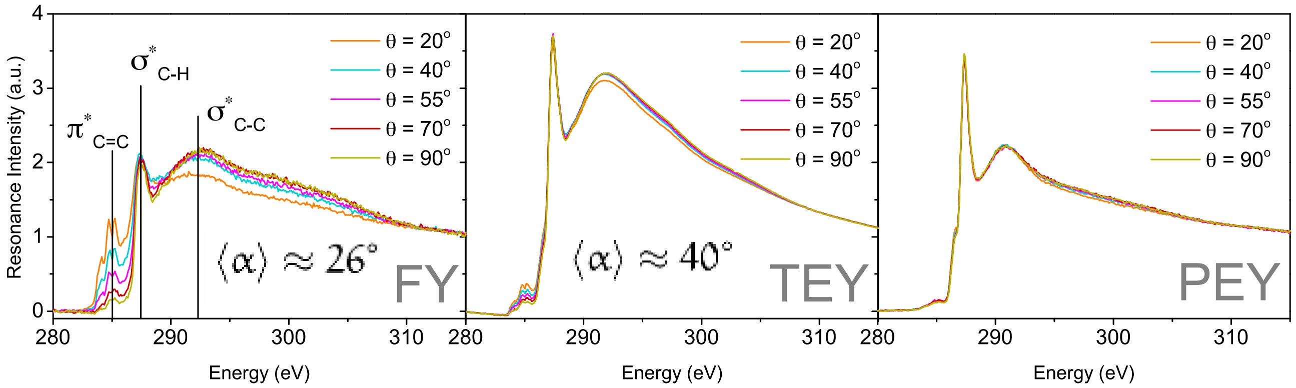

Figure S3. Partial electron yield (PEY), total electron yield (TEY) and fluorescence yield (FY) NEXAFS spectra of spin-coated film with extracted average backbone tilt angles.

D) Temperature dependent FET characteristics

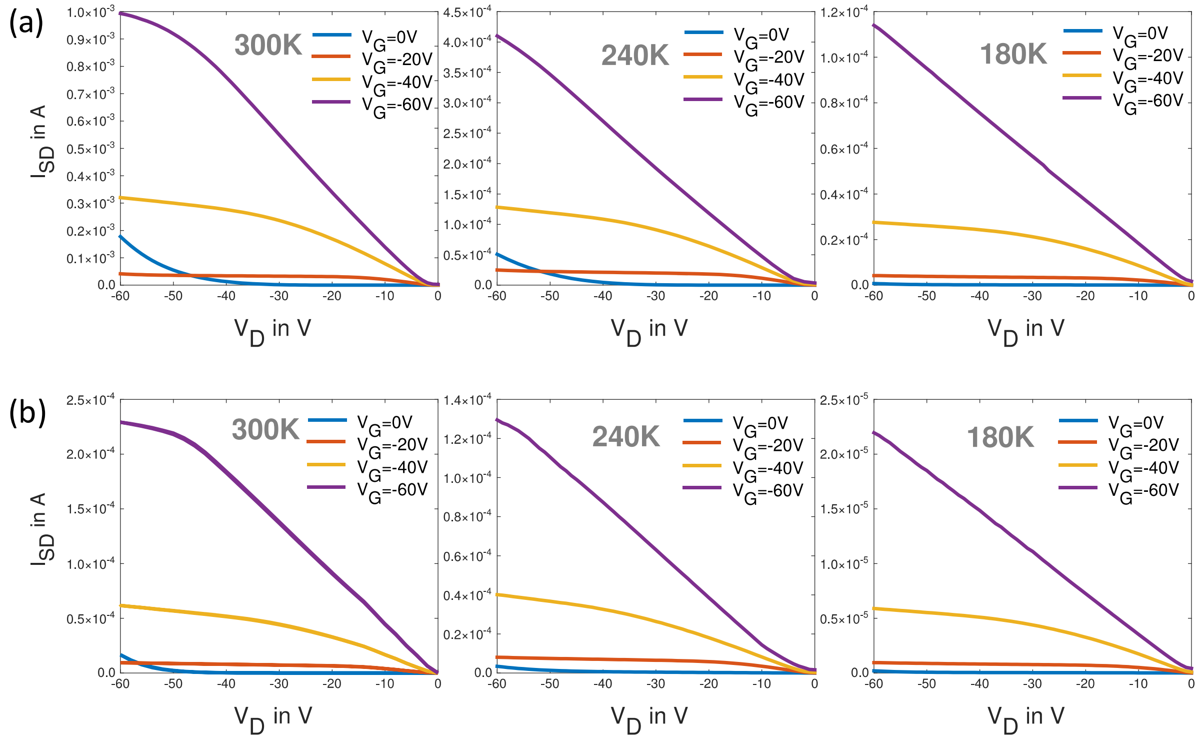

Figure S4. Output curves of FETs with channels (a) parallel and (b) perpendicular to alignment direction at 300 K, 240 K and 180 K. There is no obvious difference in contact effects between parallel and perpendicular transport at the onset of output curves. The absence of saturation at lower temperatures is commonly attributed to field-induced detrapping from shallow trap states.[8,9]

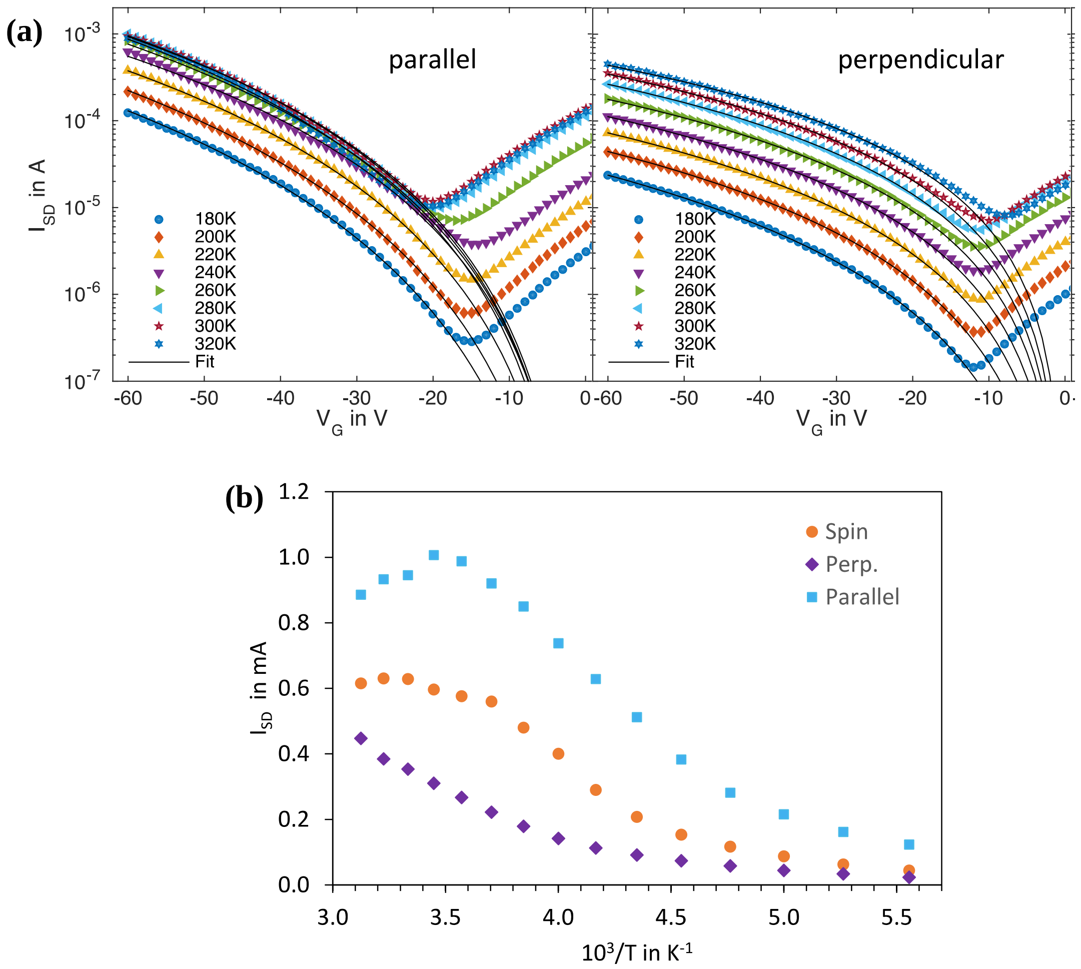

Figure S5. (a) FET saturation curves for temperatures from 180 – 320 K with fits to $I_{SD} \propto |V_G\text{-}V_{th}|^{\gamma}$. For improved visibility, only one curve for every 20 K is shown. Threshold voltages were estimated from the onset of transistor current. (b) Temperature dependence of transistor currents from saturation curves at V_G = -60 V and V_{SD} = 60 V for temperature from 180 K to 320 K in 10 K steps.

[1] J. P. Rolland, R. M. Van Dam, D. A. Schorzman, S. R. Quake, J. M. DeSimone, *J. Am. Chem. Soc.* **2004**, *126*, 2322.

[2] N. M. Kirby, S. T. Mudie, A. M. Hawley, D. J. Cookson, H. D. T. Mertens, N. Cowieson, V. Samardzic-Boban, *J. Appl. Crystallogr.* **2013**, *46*, 1670.

[3] J. Ilavsky, *J. Appl. Crystallogr.* **2012**, *45*, 324.

[4] B. C. C. Cowie, A. Tadich, L. Thomsen, in *AIP Conf. Proc.*, AIP Publishing, **2010**, pp. 307–310.

[5] B. Watts, L. Thomsen, P. C. Dastoor, *J. Electron Spectros. Relat. Phenomena* **2006**, *151*, 105.

[6] B. Watts, L. Thomsen, P. C. Dastoor, *J. Electron Spectros. Relat. Phenomena* **2006**, *151*, 208.

[7] J. Stöhr, D. A. Outka, *Phys. Rev. B* **1987**, *36*, 7891.

[8] J. H. Worne, J. E. Anthony, D. Natelson, *App. Phys. Lett.* **2010**, *96*, 53308.

[9] T. Sakanoue, H. Sirringhaus, *Nat. Mater.* **2010**, *9*, 736.
